# Risk of Cancer in Patients with Rheumatoid Arthritis Compared with the General Population: A Nationwide Cohort Study in Lithuania

**DOI:** 10.3390/medicina62020290

**Published:** 2026-02-01

**Authors:** Vaida Gedvilaitė, Augustė Kačėnienė, Gintarė Jonušienė, Jolanta Dadonienė, Dalia Miltinienė, Giedrė Deresevičienė, Giedrė Smailytė

**Affiliations:** 1 Chemotherapy Department, Medical Oncology Center, National Cancer Center, Vilnius University Hospital Santaros klinikos, 08660 Vilnius, Lithuania; 2 Laboratory of Cancer Epidemiology, National Cancer Institute, 08406 Vilnius, Lithuaniagiedre.smailyte@nvi.lt (G.S.); 3 Antakalnis Outpatient Clinic, 10207 Vilnius, Lithuania; gintare.jonusiene@gmail.com; 4 Department of Public Health, Institute of Health Sciences, Faculty of Medicine, Vilnius University, 03101 Vilnius, Lithuania; 5 Clinic of Rheumatology, Orthopaedic Traumatology and Reconstructive Surgery, Institute of Clinical Medicine, Vilnius University Faculty of Medicine, 03101 Vilnius, Lithuania; 6 Innovative Medicine Center, 08406 Vilnius, Lithuania

**Keywords:** rheumatoid arthritis, cancer, epidemiology

## Abstract

*Background and Objectives*: Rheumatoid arthritis (RA) is a multisystem autoimmune disease that needs immunosuppressive treatment. Previously, studies have shown an increased risk of cancer in patients with RA compared with the general population. The purpose of this study was to explore the associations between RA and cancer risk, providing updated insights into the incidence of specific cancers in patients with RA. *Materials and Methods*: A total of 746 cancer cases were observed, with the most common types being nonmelanoma skin cancer (139 cases), breast cancer (87 cases), lung cancer (47 cases), and Hodgkin lymphoma (43 cases). *Results:* Compared with the general Lithuanian population, patients with RA had an increased overall cancer risk, with an SIR of 1.17 and 95% CI of 1.09–1.26. Hematological cancers and nonmelanoma skin cancers were the most common types of cancer in the RA population, and patients with RA had a significantly greater risk of site-specific cancers (non-Hodgkin lymphoma: SIR 4.19, 95% CI 1.57–11.18; Hodgkin lymphoma: SIR 3.03, 95% CI 2.11–4.36; myeloma: SIR 3.00, 95% CI 1.84–4.90; leukemia: SIR 2.39, 95% CI 1.62–3.54; and skin nonmelanoma: SIR 1.54, 95% CI 1.27–1.83). Male patients with RA had an increased risk of prostate and kidney cancer (SIR 1.40, 95% CI 1.12–1.75; SIR 1.85, 95% CI 1.11–3.06). Our study revealed a significantly lower risk of colorectal cancer among patients with RA. Additionally, we observed a statistically significant reduction in the risk of mouth and pharynx cancers; however, this finding was based on only three observed cases. *Conclusions:* Patients with RA remain particularly affected by an increased cancer risk. Knowing these risks, we need clear recommendations for specific screenings in patients with RA, which could allow for early diagnosis and better cancer treatment in the early stages.

## 1. Background

Autoimmune diseases are chronic disorders that arise from a loss of immunological tolerance to self-antigens (for example, T-cell or B-cell dysfunction) [1]. Both genetic and environmental factors significantly contribute to the development and progression of autoimmune and inflammatory disorders, including rheumatoid arthritis, inflammatory bowel disease, type 1 diabetes, asthma, and coeliac disease [2]. Additionally, patients with autoimmune diseases have an increased risk of developing cancer owing to the underlying dysregulation of their immune systems or as a result of their treatments [3].

Rheumatoid arthritis (RA) is a multisystem autoimmune and inflammatory disorder. It is the most common autoimmune rheumatic disease and causes chronic pain, functional disability, morbidity, premature mortality, and a significant socioeconomic burden. While there is currently no cure for RA, early diagnosis and appropriate treatment can typically prevent joint damage and other complications.

The relationship between RA and cancer risk has been a significant focus of research and clinical interest. This is due to the autoimmune characteristics of RA, the potential shared underlying causes between rheumatic and cancer processes, and the impact of immunomodulatory therapies, which may alter the immune response and potentially increase the risk of malignancy [4].

Many population-based studies have examined overall cancer risk in patients with RA, and the findings have varied. Some studies suggest that patients with RA have a greater risk of cancer than does the general population. In contrast, other studies have indicated that their overall risk of cancer is similar to, or even lower than, that of the general population [4,5,6,7]. Meta-analyses, such as those conducted by Smitten et al. [7], have demonstrated an ~5% increased risk of cancer in patients with RA, with an increased risk of lymphoma, Hodgkin’s disease, non-Hodgkin’s lymphoma, and lung cancer, alongside a decreased risk of colorectal and breast cancers. In contrast, Chakravarty and Genovese [8] reported no overall increase in the incidence of cancer in patients with RA. These results suggest that while the overall risk of cancer in patients with RA may be similar to that in the general population, there are notable site-specific variations, with certain cancers being more prevalent and others being less prevalent in this group. Among specific malignancies, several studies have consistently reported a higher incidence of hematopoietic cancers in patients with RA, rheumatoid arthritis the risk of colorectal cancer appears to be lower [8,9,10,11]. However, research findings on the association between RA and the risk of other site-specific cancers remain diverse. The inconsistency in the reported cancer incidence rates among patients with RA highlights the need for further studies to develop a clearer understanding of these risks.

We conducted this study to explore the associations between RA and cancer risk, providing updated insights into the incidence of specific cancers in patients with RA.

## 2. Methods

### 2.1. Dataset

This study used the National Health Insurance Fund (NHIF) database to identify patients with RA. This comprehensive database includes demographic information, primary and secondary health care service records, emergency and hospital admissions, and prescriptions for reimbursed medications.

Additionally, we employed a technique involving record linkage with the Lithuanian Cancer Registry (CR) to identify cancer cases. The CR is a thorough, population-based cancer registry that has collected personal and demographic information and diagnostic data for individuals diagnosed with cancer in Lithuania since 1978.

### 2.2. Study Design and Population

In this retrospective cohort study, we aimed to investigate the relationship between RA and cancer risk. The cohort included male and female patients aged 18 years and older who had an initial diagnosis of rheumatoid arthritis (International Classification of Diseases Australian modification, ICD-10-AM diagnosis codes M05 and M06) recorded in the NHIF database between 1 January 2012, and 31 December 2019. We collected information about the prescription of glucocorticoids (prednisolone or methylprednisolone), conventional synthetic (cs) DMARDs (methotrexate, azathioprine, leflunomide, sulfasalazine, and hydrochloroquine), or bDMARDs (infliximab, etanercept, adalimumab, tocilizumab, or rituximab with available biosimilars). To prevent the risk of misclassifying rheumatoid arthritis (RA), patients who were reported to have RA and who had records of prescribed antirheumatic medications in the NHIF database were considered to have RA.

To determine the incidence of cancer within the cohort, we linked RA records to the Lithuanian National Cancer Registry using personal identification numbers assigned to all Lithuanian citizens, incorporating data up to and including 31 December 2019. We considered patients with cancer diagnoses, excluding those diagnosed with cancer before their RA diagnosis.

The person-time of observation was calculated from the date of the initial recorded RA diagnosis in the National Health Insurance Fund (NHIF) database to the date of death, emigration, or the end of the observation period (31 December 2019), whichever occurred first.

The final cohort included 11,738 patients diagnosed with RA, comprising 9398 women and 2340 men. For all patients, the data were cross-checked with data from the Health Information Center at the Institute of Hygiene to confirm their vital status and the date of death if it had been documented.

### 2.3. Statistical Analysis

The risk analysis compared the observed number of cancer cases to the expected number. The expected number of cancer cases was calculated by multiplying the total person-years observed in the cohort by sex, calendar year, and the national incidence rate specific to 5-year age groups. The observation period for each cohort member was defined as the time from the date of RA diagnosis until the cohort exit date. The cohort exit date was determined by the earliest of the following events: the date of death, emigration, or December 31, 2019. For all standardized incidence ratios (SIRs), 95% confidence intervals (95% CIs) were calculated on the basis of the assumption of a Poisson distribution for the number of observed cancer cases and deaths.

All the statistical analyses were performed via STATA 15 statistical software (StataCorp. 2020. Stata Statistical Software: Release 15.1. College Station, TX, USA).

The study was conducted in accordance with the Declaration of Helsinki, and the Vilnius Regional Biomedical Research Ethics Committee (No. 158200-17-958-462) approved the protocol.

## 3. Results

During the study period, 11,738 patients with RA accounted for a total of 64,887 person-years for the main analysis. The characteristics of the patients with RA are outlined in Table 1. There were significantly more women than men in the study group, accounting for 68.6% and 31.4%, respectively. The median age at the time of inclusion in the study was 58.69 years. Approximately 28% of patients had biologic exposure (anti-TNF/IL-6 inhibitors), vs. 72% csDMARDs only.

A total of 746 cancer cases were observed, with the most common types being nonmelanoma skin cancer (139 cases), breast cancer (87 cases), lung cancer (47 cases), and Hodgkin lymphoma (43 cases). The SIRs by cancer type and sex can be found in Table 2. Compared with the general Lithuanian population, patients with RA had an increased overall cancer risk, with an SIR of 1.17 and 95% CI of 1.09–1.26.

Hematological cancers and nonmelanoma skin cancers were the most common types of cancer in the RA population, and patients with RA had a significantly greater risk of site-specific cancers (non-Hodgkin lymphoma: SIR 4.19, 95% CI 1.57–11.18; Hodgkin lymphoma: SIR 3.03, 95% CI 2.11–4.36; myeloma: SIR 3.00, 95% CI 1.84–4.90; leukemia: SIR 2.39, 95% CI 1.62–3.54; and skin nonmelanoma: SIR 1.54, 95% CI 1.27–1.83). Male patients with RA had an increased risk of prostate and kidney cancer (SIR 1.40, 95% CI 1.12–1.75; SIR 1.85, 95% CI 1.11–3.06).

Our study revealed a significantly lower risk of colorectal cancer among patients with RA. The SIRs were 0.62 for colon cancer (95% CI 0.41–0.96) and 0.55 for rectal cancer (95% CI 0.32–0.94). Additionally, we observed a statistically significant reduction in the risk of mouth and pharynx cancers; however, this finding was based on only three observed cases.

## 4. Discussion

This observational study revealed an increased overall cancer risk among patients with RA compared with the general population. DAS28 scores were unavailable in the administrative NHIF/LCR databases used, which capture ICD-10 diagnoses, prescriptions, and procedures but not granular clinical metrics like DAS28. This excess risk was essentially carried by men, who presented a 26% increased risk of any cancer, whereas women demonstrated a comparatively smaller increased relative risk, at 14%. This increased overall cancer risk among patients with RA is in line with the results of previous studies [4,5,6,7,8,9]. Certain site-specific cancers were overrepresented among Lithuanian patients with RA. We observed an increased risk of hematologic malignancies and nonmelanoma skin cancers in both sexes, as well as increased risks of prostate and kidney cancers in males. The risks for other cancers were not increased, and the risk of colorectal cancer was lower than expected in the general population. The inflammatory processes associated with RA can disrupt immune function or affect the effectiveness of immunosuppressive treatments. This disruption may increase the risk of developing lymphoma or other hematological malignancies.

Smoking and socioeconomic status (SES) are key unmeasured confounders; Lithuania’s high smoking prevalence likely contributes to lung/leukemia excess, while lower SES may drive kidney cancer. Surveillance bias is acknowledged for skin/hematologic cancers due to rheumatology monitoring. One of the reasons why cancers diagnosed shortly after RA diagnosis could be that patients were actively followed up by their doctors.

The risk factors and pathways of lymphoma complicating RA are not well understood. The risk of lymphoma is 1.5-fold to 3-fold greater in patients with RA than in the overall population [12]. An increased risk of Hodgkin’s lymphoma or non-Hodgkin’s lymphoma in both sexes was found in the patients with RA in our study. In a French case-control study, lymphomas complicating RA were mostly diffuse large B-cell lymphomas (n = 27, 50.0%). The risk of lymphoma in patients with RA in this study increased with increasing disease activity and severity [9]. In a Swedish cohort study, the average lymphoma risk was approximately 50% higher in patients with RA than in the general population. The results of this study suggest that treatment with biologic disease-modifying antirheumatic drugs does not further increase the already increased risk of lymphoma in patients with RA [10]. The risk of Hodgkin’s lymphoma is frequently associated with Epstein–Barr virus (EBV) and immunosuppressive treatment for RA [9]. The diagnosis of multiple myeloma is often delayed due to a lack of recognition of the most common presenting symptoms, and multiple myeloma can be misdiagnosed as RA. Some cases demonstrate that multiple myeloma can mimic seropositive RA, presenting as cutaneous amyloid nodules that may be mistaken for RA nodules. Additionally, various hematological malignancies can sometimes resemble rheumatic syndromes [12,13]. Our research revealed multiple myeloma in one man and sixteen women, with an overall SIR of 2.47 (95% CI 1.53–3.97). A study by Luo et al. [14] indicated that patients with RA had a greater risk of developing leukemia, with an SIR of 1.51 (95% CI 1.34–1.70). Our study revealed similar results, revealing an even greater risk of leukemia, with an SIR of 2.35 (95% CI 1.68–3.29).

In their meta-analysis, Li et al. [15] reported that RA reduced the risk of developing colorectal cancer, with an SIR of 0.69 (95% CI 0.53–0.85). This effect is caused by immune-mediated inflammation, with IL6R being a key regulatory gene [15]. We identified a negative causal relationship between RA and the risk of colorectal cancer (SIR 0.62, 95% CI 0.41–0.96). Furthermore, the use of nonsteroidal anti-inflammatory drugs (NSAIDs) by patients with RA might lead to a reduced risk of colorectal cancer, although the exact mechanism behind this effect is not yet fully understood.

Our study revealed an SIR of 1.52 (95% CI 1.28–1.79) for nonmelanoma skin cancer among patients with RA. An increased risk of nonmelanoma skin cancer has also been reported in other studies [16,17]. One of the possible causes was reported by Wang et al. [17], who demonstrated that patients with RA receiving TNF antagonists had an increased risk of developing nonmelanoma skin cancer in a systematic review and meta-analysis [16].

Wheeler et al. [18] conducted a study on veterans with and without RA to determine whether RA affects the risk of developing prostate cancer. Their findings indicated that RA was modestly associated with a higher risk of prostate cancer, with an adjusted odds ratio of 1.12 (95% CI 1.04–1.20). Additionally, a meta-analysis of 17 studies revealed that the overall SIR of prostate cancer in patients with RA was 1.15 (95% CI 0.98–1.34) compared with that in the general population [18]. Our study further demonstrated an increased risk of prostate carcinoma in individuals with RA, with an SIR of 1.4 and 95% CI of 1.12–1.75, compared with the general population.

In a French study, kidney cancer was found to be more common among patients with RA [5]. In the UK, there was an increase in the hazard ratio for kidney cancer associated with RA in women, with a hazard ratio of 1.30 (95% CI 1.16–1.45) [19]. Additionally, our findings indicated that the risk of kidney cancer was increased in male patients with RA, with an SIR of 1.85 (95% CI 1.11–3.06), whereas no increase in risk was observed among female patients.

In our study, we found that the risks of lung, breast, and cervical cancer were not increased in patients with RA. However, the literature presents conflicting results. For example, the study by Yang et al. [19] investigated RA-associated cancer risks in a large cohort of approximately 1.3 million women in the UK. A modest decrease in the risk of breast cancer (HR 0.96, 95% CI 0.93–0.99) and an increased risk of lung cancer (HR 1.21, 95% CI 1.15–1.26) were reported [10]. In another study by Beydon et al. [5], breast and endometrial cancers were found to be less common in patients with RA than in the general population, with an SIR of 0.91 (95% CI 0.88–0.94) for breast cancer and an SIR of 0.77 (95% CI 0.71–0.84) for endometrial cancer. A meta-analysis revealed no association between RA and cervical cancer risk, reporting 297 cases across 15 studies and an SIR of 0.87 (95% CI 0.72–1.05) [7]. Our analysis yielded a similar finding, with an SIR of 0.87 and 95% CI of 0.54–1.43. However, according to the publication by Yang et al. [19], there was an increased risk of RA linked to cervical cancer (HR 1.39, 95% CI 1.11–1.75). The authors hypothesized that RA may be associated with a higher rate of general infections and with human papillomavirus (HPV) infections, which can lead to cervical cancer [10].

Interestingly, we observed a decreased risk of mouth and pharynx cancer, colon cancer, and lower intestinal tract carcinoma in patients with RA compared with the general population.

This study covered the entire population of Lithuania, and 11,738 patients with RA were included. Our cohort primarily captures prevalent RA cases from registry entry, as first-year observations would predominantly reflect pre-existing RA diagnosis. However, owing to the small numbers of patients with some site-specific cancers, the associated cancer risk figures should be interpreted with caution. Another important limitation is that only age and sex were considered in the risk assessment, which may affect the comprehensiveness of the findings. Patients treated with disease-modifying drugs for autoimmune inflammatory diseases, such as rituximab or abatacept, are known to have the highest risk of cancer [5]. However, our research did not evaluate the potential role of specific treatments received by patients.

## 5. Conclusions

Patients with RA remain particularly affected by an increased cancer risk. RA was associated with elevated site-specific cancer risks, warranting vigilant screening. We observed an increased risk of hematologic malignancies and nonmelanoma skin cancers in both sexes, as well as increased risks of prostate and kidney cancers in males. In contrast, the risk of colorectal cancer was reduced. Knowing these risks, we need clear recommendations for specific screenings in patients with RA, which could allow for early diagnosis and better cancer treatment in the early stages.

## Figures and Tables

**Table 1:** Characteristics of the study population.

Patient Characteristics	Male	%	Female	%	Total	%
Number of patients	2340	19.94	9398	80.06	11,738	100.00
Person-years	11,772.01		53,115.25		64,887.26	
Mean age at diagnosis, years (±SD)	58.38 (14.29)		58.77 (14.21)		58.69 (14.22)	
Age at RA diagnosis, years						
<40	247	10.56	924	9.83	1171	9.98
40–50	360	15.38	1372	14.60	1732	14.76
50–59	634	27.09	2755	29.31	3389	28.87
60–69	576	24.62	2107	22.42	2683	22.86
≥70	523	22.35	2240	23.83	2763	23.54
Types of cancer per subject						
0	2118	90.51	8907	94.78	11,025	93.93
1	210	8.97	472	5.02	682	5.81
≥2	12	0.51	19	0.20	31	0.26
Exposure to biological therapy						
Yes	189	8.08	726	7.73	915	7.80
No	2151	91.92	8672	92.27	10,823	92.20

**Table 2:** Numbers of new cancer cases and SIR among patients with RA during 2012–2019.

Diagnosis	ICD-10	Male					Female					Overall				
		Obs	Exp	SIR	95% CI		Obs	Exp	SIR	95% CI		Obs	Exp	SIR	95% CI	
All Sites	C00–C96	234	185.03	1.26	1.11	1.44	512	450.42	1.14	1.04	1.24	746	635.45	1.17	1.09	1.26
Lip	C00	0	0.31	-	-	-	0	0.34	-	-	-	0	0.66	-	-	-
Mouth and pharynx	C01–C14	2	6.01	0.33	0.08	1.33	1	4.69	0.21	0.03	1.51	3	10.70	0.28	0.09	0.87
Esophagus	C15	2	3.79	0.53	0.13	2.11	1	1.88	0.53	0.07	3.77	3	5.67	0.53	0.17	1.64
Stomach	C16	8	9.67	0.83	0.41	1.65	16	17.72	0.90	0.55	1.47	24	27.39	0.88	0.59	1.31
Other digestive organs	C17, C26, C48	0	0.59	-	-	-	2	2.29	0.87	0.22	3.50	2	2.88	0.70	0.17	2.78
Colon	C18	7	9.00	0.78	0.37	1.63	14	24.69	0.57	0.34	0.96	21	33.69	0.62	0.41	0.96
Rectum, rectosigmoid, anus	C19–C21	4	7.46	0.54	0.20	1.43	9	16.39	0.55	0.29	1.06	13	23.85	0.55	0.32	0.94
Liver	C22	10	3.08	3.24	1.74	6.03	4	4.66	0.86	0.32	2.29	14	7.74	1.81	1.07	3.05
Gallbladder, bile ducts	C23, C24	1	0.93	1.07	0.15	7.61	0	4.07	-	-	-	1.00	5.00	0.20	0.03	1.42
Pancreas	C25	5	5.37	0.93	0.39	2.24	18	15.77	1.14	0.72	1.81	23	21.14	1.09	0.72	1.64
Nasal cavity, middle ear, accessory sinuses	C30, C31	0	0.31	-	-	-	0	0.70	-	-	-	0	1.01	-	-	-
Larynx	C32	0	3.58	-	-	-	0	0.73	-	-	-	0	4.31	-	-	-
Lung, trachea	C33, C34	27	25.15	1.07	0.74	1.57	20	18.20	1.10	0.71	1.70	47	43.35	1.08	0.81	1.44
Bone and connective tissue	C40, C41, C45–C47, C49	1	0.92	1.09	0.15	7.72	6	3.05	1.97	0.89	4.39	7	3.97	1.77	0.84	3.70
Skin, melanoma	C43	3	2.46	1.22	0.39	3.78	12	10.53	1.14	0.65	2.01	15	12.99	1.15	0.70	1.92
Skin, nonmelanoma	C44	26	17.46	1.49	1.01	2.19	113	74.27	1.52	1.27	1.83	139	91.73	1.52	1.28	1.79
Breast	C50	0	0.27	-	-	-	87	88.27	0.99	0.80	1.22	87	88.54	0.98	0.80	1.21
Vulva	C51	-	-	-	-	-	1	3.06	0.33	0.05	2.32	-	-	-	-	-
Other female and male genital organs	C52, C57–C58, C60, C63	0	0.56	-	-	-	3	2.05	1.46	0.47	4.53	-	-	-	-	-
Cervix uteri	C53	-	-	-	-	-	16	18.29	0.87	0.54	1.43	-	-	-	-	-
Corpus uteri	C54, C55	-	-	-	-	-	43	38.61	1.11	0.83	1.50	-	-	-	-	-
Ovary	C56	-	-	-	-	-	19	21.09	0.90	0.57	1.41	-	-	-	-	-
Prostate	C61	78	55.74	1.40	1.12	1.75	-	-	-	-	-	-	-	-	-	-
Testis	C62	0	0.19	-	-	-	-	-	-	-	-	-	-	-	-	-
Kidney	C64	15	8.12	1.85	1.11	3.06	17	15.90	1.07	0.66	1.72	32	24.03	1.33	0.94	1.88
Other urinary tract	C65, C66, C68	2	0.54	3.69	0.92	14.77	0	1.19	-	-	-	2	1.73	1.16	0.29	4.63
Urinary bladder	C67	4	6.54	0.61	0.23	1.63	6	4.62	1.30	0.58	2.89	10	11.16	0.90	0.48	1.67
Eye and adnexa	C69	0	0.28	-	-	-	2	0.92	2.18	0.55	8.72	2	1.19	1.68	0.42	6.70
Central nervous system	C70–C72	2	2.36	0.85	0.21	3.39	9	7.38	1.22	0.63	2.34	11	9.73	1.13	0.63	2.04
Thyroid	C73	2	0.82	2.43	0.61	9.73	12	12.91	0.93	0.53	1.64	14	13.74	1.02	0.60	1.72
Other endocrine organs	C74, C75	0	0.15	-	-	-	1	0.39	2.59	0.37	18.42	1	0.53	1.87	0.26	13.28
Other and ill-defined sites	C76–C80	7	4.60	1.52	0.72	3.19	6	9.31	0.64	0.29	1.43	13	13.92	0.93	0.54	1.61
Non-Hodgkin lymphoma	C81	1	0.20	4.90	0.69	34.77	3	0.75	4.00	1.29	12.41	4	0.95	4.19	1.57	11.18
Hodgkin lymphoma	C82–C85	14	2.84	4.93	2.92	8.32	29	9.57	3.03	2.11	4.36	43	12.41	3.46	2.57	4.67
Other and unspecified malignant neoplasms of lymphoid, hematopoietic and related tissue	C88, C96	2	0.12	17.01	4.25	68.01	0	0.34	-	-	-	2.00	0.46	4.39	1.10	17.54
Myeloma	C90	1	1.57	0.64	0.09	4.53	16	5.33	3.00	1.84	4.90	17	6.89	2.47	1.53	3.97
Leukemia	C91–C95	9	4.03	2.23	1.16	4.29	25	10.45	2.39	1.62	3.54	34	14.48	2.35	1.68	3.29

## Data Availability

The original contributions presented in this study are included in the article. Further inquiries can be directed to the corresponding author.
